# Oncologists’ communication about tobacco and alcohol use during treatment for esophagogastric cancer: a qualitative observational study of simulated consultations

**DOI:** 10.1007/s00520-024-08847-y

**Published:** 2024-09-20

**Authors:** Danique W. Bos-van den Hoek, Loïs F. van de Water, Pieter G. Vos, Meeke Hoedjes, Ruud Roodbeen, Bastiaan R. Klarenbeek, Debby Geijsen, Ellen M. A. Smets, Hanneke W. M. van Laarhoven, Inge Henselmans

**Affiliations:** 1 Department of Medical Psychology, Amsterdam UMC Location University of Amsterdam, Amsterdam, the Netherlands; 2 Department of Medical Oncology, Amsterdam UMC Location University of Amsterdam, Amsterdam, the Netherlands; 3 Amsterdam Public Health, Quality of Care, Amsterdam, the Netherlands; 4 Cancer Center Amsterdam, Cancer Treatment and Quality of Life, Amsterdam, the Netherlands; 5 Department of Medical and Clinical Psychology, Center of Research On Psychological Disorders and Somatic Diseases, Tilburg University, Tilburg, the Netherlands; 6 Department of Health Promotion, Maastricht University, Maastricht, the Netherlands; 7 Department of Surgery, Radboud University Medical Center, Nijmegen, the Netherlands; 8 Department of Radiation Oncology, Amsterdam UMC Location University of Amsterdam, Amsterdam, the Netherlands

**Keywords:** Cancer, Communication, Health behavior, Smoking, Alcohol, Prevention

## Abstract

**Purpose:**

Tobacco and alcohol use influence cancer risk as well as treatment outcomes, specifically for esophageal and gastric cancer patients. Therefore, it is an important topic to discuss during consultations. This study aims to uncover medical, radiation, and surgical oncologists’ communication about substance use, i.e., tobacco and alcohol use, in simulated consultations about curative and palliative esophagogastric cancer treatment.

**Methods:**

Secondary analyses were performed on *n* = 40 standardized patient assessments (SPAs) collected in three Dutch clinical studies. Simulated patients with esophagogastric cancer were instructed to ask about smoking or alcohol use during treatment. The responses of the 40 medical, radiation, and surgical oncologists were transcribed verbatim, and thematic analysis was performed in MAXQDA.

**Results:**

Oncologists consistently advocated smoking cessation during curative treatment. There was more variation in their recommendations and arguments in the palliative compared to the curative setting and when addressing alcohol use instead of smoking. Overall, oncologists were less stringent regarding behavior change in the palliative than in the curative setting. Few oncologists actively inquired about the patient’s perspective on the substance use behavior, the recommended substance use change, or the support offered.

**Conclusion:**

Clear guidelines for oncologists on when and how to provide unequivocal recommendations about substance use behavior change and support to patients are needed. Oncologists might benefit from education on how to engage in a conversation about smoking or alcohol.

**Supplementary Information:**

The online version contains supplementary material available at 10.1007/s00520-024-08847-y.

## Introduction

It is widely recognized that lifestyle behavior influences the risk of developing cancer and affects treatment outcomes, such as toxicities, cancer recurrence, and overall survival [1–5], as well as the risk of comorbidities, such as cardiovascular disease [6–9]. Among these lifestyle behaviors are smoking tobacco and drinking alcohol [8, 10–12]. Cancer patients smoking tobacco generally experience not only increased morbidity, toxicity, complications, and hospitalization, but also decreased performance status, survival, and lower quality of life [13–23]. Besides, there is an increased risk for second primary cancer [24]. There is also tentative evidence for the influence of alcohol use on cancer treatment outcomes, such as survival, progression of disease, and cancer recurrence [25–29]. Nevertheless, after a cancer diagnosis, still many patients continue to smoke and drink alcohol [30–33]. Some patients do show intentions, attempts, and success in changing their behavior, especially when diagnosed with cancer types related to tobacco and alcohol use [34, 35].

Advice or support of the healthcare provider is often recognized as playing a pivotal role in the success of health behavior change [35, 36]. The oncologist may even be in an optimal position for promoting and supporting health behavior change, when capitalizing on the “teachable moment” created by the cancer diagnosis [35]. Healthcare providers in cancer care generally agree on the importance of addressing health behavior [37–39]. For tobacco use in particular, oncologists agree that smoking negatively influences treatment outcomes in both the curative and palliative setting and that smoking cessation should be a standard part of treatment [40–42]. Oncologists report to ask or advise smokers to quit, yet do not often provide cessation support [40–42]. They experience several barriers, including their perceived inability to get patients to quit, patients’ resistance, a lack of time for counseling, and a lack of training in cessation interventions [35, 40, 43]. Less is known about oncologists’ perspectives on alcohol consumption and their role in addressing patients’ drinking habits.

The use of tobacco and alcohol is associated with the development of esophageal and gastric cancer as well as worse survival rates in these types of cancers [25, 44–50]. Hence, informing esophagogastric cancer patients on the possible consequences of tobacco and alcohol use, and offering them advice and support in quitting or reducing their use, is particularly relevant. Besides oncologists’ self-reports on their role in smoking cessation, it is unclear if and how oncologists actually communicate about either alcohol or tobacco use in the consultation room. The current study aims to gain insight into oncologists’ communication about substance use, i.e., tobacco and alcohol use, in simulated consultations about curative and palliative esophagogastric cancer treatment. More specifically, the study aims to examine what advice oncologists provide, what arguments they use, and what support they offer. It furthermore aims to explore the way oncologists communicate about smoking and drinking behavior, e.g., how oncologists phrase their recommendations.

## Methods

### Study design

In this secondary analysis, data from three different Dutch clinical studies were used. In these projects, training programs for surgical, radiation, and medical oncologists on shared decision-making (SDM) [51–54] were evaluated. Evaluation took place by assessing audio-recorded or video-recorded standardized patient assessments (SPAs), conducted before and after training. The standardized patients were instructed to ask about either smoking or alcohol use during cancer treatment. In the current study, a qualitative observational study design was adopted to analyze the fragments that focused on smoking or alcohol use.

### SPAs

In the SPAs, a simulated consultation between an oncologist and an actor (standardized patient) took place. The sample consisted of medical, surgical, and radiation oncologists (staff or in training) working in Dutch (academic) hospitals. In addition, one nurse specialist and one physician assistant were part of the sample. Prior to the SPA, oncologists, who were aware of the simulated nature of the SPA, received a simulated medical file. A multidisciplinary team of psychologists and oncologists developed scripts for the standardized patients. The patient cases concerned incurable disease for medical oncologists (palliative setting) and curable disease for radiation and surgical oncologists (curative setting). Three male actors played patients with either esophageal or gastric cancer who needed to decide on starting treatment. They were instructed to ask the oncologist briefly about alcohol use (“Can I continue drinking alcohol?”) or smoking (“Can I continue smoking?”) during treatment. The SPAs took place in-person or online due to COVID-19 restrictions. See Supplementary File 1 for more information about the SPA cases.

### Fidelity

In the SPAs, actors were allowed to use their own words for posing the questions and not instructed on how to respond to the answer. Sometimes, actors deviated from the script on the number of cigarettes/drinks they consumed. Or they inquired about both alcohol and tobacco use in the same consultation. In the latter cases, we only analyzed the responses to the question that the actor was intended to ask.

### Study procedures

Forty SPAs were randomly selected for inclusion in the current study, from a total of 95 SPAs with untrained oncologists (pre-intervention or control group). An even distribution was ensured between the palliative setting (SPAs of medical oncologists) and curative setting (SPAs of surgical and radiation oncologists) and between SPAs including inquiries about alcohol use and smoking.

### Analysis

The relevant fragments from the video-recorded SPAs were identified and transcribed verbatim by PV (medical doctor in training). Two researchers (PV and IH (assistant professor of Medical Psychology)) read and coded the transcripts independently in MAXQDA 2022 using thematic analysis [55]. After each set of *n* = 10 SPAs, they discussed discrepancies to reach a consensus and develop a coding scheme. After two such coding rounds, the provisional coding scheme was discussed with LW (psychologist) and DB (health scientist), and some small adjustments were made. Subsequently, PV and IH coded the last two sets of *n* = 10 SPAs, which demonstrated data saturation, i.e., no further adjustments to the coding tree. After initial coding, IH, LW, and DB re-read the fragments and renamed, merged, or deleted codes to reach the final coding tree. Consensus was reached through discussion and adjustments were incorporated in the coding of the full set.

## Results

Thirty-nine SPAs were included, as one selected SPA did not contain an actor patient’s question about alcohol use or smoking and was excluded from the analyses. See Table 1 for further details.

**Table 1:** Participant characteristics (*N* = 39)

Age (in years, mean (range))	45.2 (34–63)
Sex, *n* (%), female	19 (48.7)
Hospital, *n* (%), academic	22 (56.4)
Setting of SPA and discipline, *n* (%)	
Palliative	20 (51.2)
Medical oncology	20 (51.2)
Curative	19 (48.7)
Radiation oncology	9 (23.1)
Surgical oncology	10 (25.6)
Setting of SPA and substance type, *n (%)*	
Palliative	
Smoking	10 (25.6)
Alcohol	10 (25.6)
Curative	
Smoking	10 (25.6)
Alcohol	9 (23.1)
Position, *n* (%)	
Oncologist	34 (87.2)
Oncologist in training	3 (7.7)
Clinical nurse specialist/physician assistant^a^	2 (5.1)

Abbreviation: *SPA* standardized patient assessment

^a^Two non-MDs were included in the sample. For the sake of clarity, we nevertheless refer to the sample as “oncologists”

### Substance use behavior advice

#### Overall

Of all 39 oncologists, *n* = 21 recommended standardized patients to change their substance use behavior, of which *n* = 13 advised patients to quit and *n* = 8 to moderate substance use. A total of *n* = 18 oncologists did not specifically recommend a change, of whom some made general remarks about the undesirability of the behavior, and *n* = *6* mentioned moderate use is generally preferable. For a subset of consultations, the categorization was complex as the oncologist’s advice was ambiguous. For example, the oncologist would mention arguments for a change in smoking or alcohol use, yet would not conclude a change would be advisable. Or the oncologist would state that it would be best to change the behavior while at the same time stating that it was not strictly required to do so in the context of treatment (Table 2, Q1).

**Table 2:** Illustrative quotes

	Substance	Oncologist	Setting	Quote
Q1	Smoking	Medical oncologist	Palliative	Patient: I smoke half a pack a day, can I, during the chemo, if I do it, can I keep doing that? Medical oncologist: Well, of course, smoking in general, right, is not healthy. And that’s pretty much my answer. So, it would be good, also for extra lung capacity (..) to at least cut back. But yes, if you ask me (…) are there any objections considering the chemo, of course not. But regarding your general health and to ensure good lung function, then it would be worth considering. Strictly speaking, there are no objections Patient: OK
Q2	Smoking	Surgical oncologist	Curative	Surgical oncologist: But smoking as well, we know that smoking increases those complications, the chance of those happening. We know from research that quitting six weeks before surgery, that it will make a significant difference. That the chance of pneumonia, but also the chance of anastomotic leakage, that that clearly diminishes
Q3	Smoking	Surgical oncologist	Curative	Surgical oncologist: (..) it’s really easy to just say ‘quit.’ For a number of reasons it would greatly benefit you. The effectiveness of the chemo and radiation therapy will be a lot better when you don’t smoke, that’s because of the nicotine, nicotine constricts the smaller blood vessels, which is exactly where chemo and radiation need to do their work, so the effectiveness will increase a great deal
Q4	Alcohol	Medical oncologist	Palliative	Medical oncologist: I’ve spoken to people who do it/keep doing it and say it doesn’t bother them, and I see people who say it affects their taste to such an extent that they really (..) don’t feel like alcohol and prefer to drink other things.
Q5	Smoking	Medical oncologist	Palliative	Medical oncologist: So, I think that at this point that that is more important for you than (..) to force you to quit smoking. Especially since we’re in a life-extending stage, where your quality of life plays a bigger part than saying: we’re going to quit smoking. You’ll probably read, when you’re looking for information online or in books, that smoking possibly weakens the effect of chemo, but between you and me, it’s more important to me that you maintain a good quality of life. And that means that if it helps you, then I’m OK with you continuing to smoke.
Q6	Smoking	Medical oncologist	Palliative	Medical oncologist: I don’t think there’s a definitive reason to quit smoking necessarily. In general, I think smoking is unhealthy… Patient: No, I… Yeah… Medical oncologist: But there is no definitive reason for you to not be allowed to smoke during the chemo. Patient: OK. Well, I’ll try.
Q7	Smoking	Surgical oncologist	Curative	Surgical oncologist: Yes. So, the question would be whether you know what things could help you, because there are a lot of options. We could have you join a stop smoking program at an outpatient clinic, or see your GP, who could support you in this. Sometimes it’s hard to imagine what could work for you and it could also be a good idea to talk to your GP about that, discuss the options. It might not be a bad idea, regardless, especially considering all of the treatment options, to talk this through with the GP as well and maybe also discuss the smoking.
Q8	Smoking	Surgical oncologist	Curative	Surgical oncologist: And it’s not something you have to do alone, there are people to support you, because it’s not easy at all, especially in this stressful time Patient: Right, I was going to say that, yes, sometimes it’s a bit of an escape, it gives me a grip on things… Surgical oncologist: Yes, I can imagine that, but it really helps bring down the risk of complications. So I always tell my patients: we’re going to do this together and we’re going to fight this together, we’re really going to give it our all to try and make you better.
Q9	Smoking	Surgical oncologist	Curative	Surgical oncologist: Well, yes, I, as a surgeon, of course am going to try and explain to you why I think you should quit. […] you’re old enough to know and decide what you want to do, but of course I have an opinion about this.
Q10	Smoking	Physician assistant (PA) surgical oncology	Curative	PA: The thing is, when you quit, you’ll have to quit without substitutes, without patches or gum, because they contain nicotine and it has the same effect. Most people succeed, in here, to quit. It’s your last chance and you need to…, if you choose to fully cooperate: diet, exercise, no smoking.
Q11	Alcohol	Medical oncologist	Palliative	Patient: That’s my question, too: can I still do that once I start the chemo, can I drink alcohol during the chemo? Medical oncologist: It would be best if you didn’t, I don’t think you’ll feel like it those first few weeks, maybe the second week once you’ve started on the tablets Officially, you’re allowed though, I mean I’m not very strict, I think it’s about your quality of life. So I’d say: limit yourself, and watch, listen to your body. When you get nauseous, then maybe it wasn’t a good idea. I think that’s the way to go about it.
Q12	Smoking	Radiation oncologist	Curative	Patient: Sometimes I don’t finish it, you know (ed: half a pack), and I wish I could go without it, but these last few days I notice it’s a kind of an escape, a kind of anchor, yeah, it sounds really, erm, yeah, it sounds… Radiation oncologist: Yes, it makes sense, it’s a really stressful time, of course. Patient: Yes. Radiation oncologist: And that’s when it’s hard to quit.

#### Setting and substance

In the curative setting (*n* = 19), most oncologists (*n* = 16/19) recommended patients to change their behavior during treatment. In this setting, all smokers (*n* = 10) were advised to change: the majority to quit (*n* = 9/10) and one to moderate (*n* = 1/10). Oncologists were less strict in consultations about alcohol use (*n* = 9). Advice varied between quitting alcohol use (*n* = 3/9), moderation (*n* = 3/9), and no change at all (*n* = 3/9).

In the palliative setting (*n* = 20), most oncologists (*n* = 15/20) did not explicitly recommend a behavior change. Some oncologists advised to change behavior regarding alcohol use (*n* = 3/5) and others regarding smoking (*n* = 2/5). Mostly, they advised to moderate instead of quitting.

### Arguments

Tables 3 and 4 show the arguments provided by oncologists to back up either their advice to change substance use behavior or not. In some cases, arguments were not in line with the eventual advice (Tables 3 and 4). This was more frequent in the palliative setting than in the curative setting (*n* = 16 vs. *n* = 2).

**Table 3:** Overview of provided arguments to support advice

Advice, setting, and substance	Total number of SPAs	Number of arguments provided to support advice								Total arguments for advice	Total arguments mentioned
		Advice to change						No advice to change			
		Lifestyle is unhealthy in general	Changed taste or desire during treatment	Risk of complications or side effects	Negative impact on treatment effect	Negative impact on physical condition during treatment in general	Making use of the opportunity	Quality of life is all that matters	No impact on treatment effects		
**Change**	**21**	**6**	**5**	**10**	**4**	**6**	**2**	**0**	**0**	**33**	**34**
** Curative**	**16**	**4**	**3**	**9**	**4**	**6**	**2**			**28**	**28**
Alcohol	6	1	3	1	0	1	0			6	6
Smoking	10	3	0	8	4	5	2			22	22
** Palliative**	**5**	**2**	**2**	**1**	**0**	**0**	**0**			**5**	**6**
Alcohol	3	0	2	0	0	0	0			2	3
Smoking	2	2	0	1	0	0	0			3	3
**No change**	**18**	**0**	**0**	**0**	**0**	**0**	**0**	**7**	**3**	**10**	**27**
** Curative**	**3**							**0**	**1**	**1**	**3**
Alcohol	3							0	1	1	3
** Palliative**	**15**							**7**	**2**	**9**	**24**
Alcohol	7							2	0	2	9
Smoking	8							5	2	7	15

Some oncologists mentioned arguments that did not match their eventual advice (change or no change) that is why there is a separate column for total arguments for advice and total arguments mentioned

“No advice to change” means oncologists did not specifically recommend a change, of whom some made general remarks about the undesirability of the behavior or mentioned moderate use is generally preferable

**Table 4:** Overview arguments per standardized patient assessment

Setting and substance	Number of SPA		Advice	Arguments for advice								Total arguments for advice	Total arguments mentioned
				**Change**						**No change**			
				Substance use is unhealthy in general	Changed taste or desire during treatment	Risk of complications or side effects	Negative impact on treatment effect	Negative impact on physical condition during treatment in general	Making use of the opportunity	Quality of life is all that matters	No impact on treatment effects		
Curative													
Smoking	01	Change				X	X					2	2
	02	Change				X	X		X			3	3
	03	Change		X		X						2	2
	04	Change				X						1	1
	05	Change		X		X	X					3	3
	06	Change					X	X				2	2
	07	Change				X		X				2	2
	08	Change		X				X	X			3	3
	09	Change				X		X				2	2
	10	Change				X		X				2	2
Alcohol	11	Change			X	X						2	2
	12	No change			O						X	1	2
	13	Change			X							1	1
	14	Change			X							1	1
	15	No change			O							0	1
	16	Change		X								1	1
	17	No change										0	0
	18	Change										0	0
	19	Change						X				1	1
Palliative													
Smoking	20	No change		O								0	1
	21	No change		O								0	1
	22	No change								X	X	2	2
	23	Change		X								1	1
	24	No change		O				O				0	2
	25	Change		X		X						2	2
	26	No change		O						X		1	2
	27	No change			O		O			X		1	3
	28	No change			O					X	X	2	3
	29	No change								X		1	1
Alcohol	30	Change			X					O		1	2
	31	No change										0	0
	32	No change			O					X		1	2
	33	No change			O			O				0	2
	34	No change			O							0	1
	35	No change				O						0	1
	36	No change			O					X		1	2
	37	Change										0	0
	38	Change			X							1	1
	39	No change			O							0	1

X = arguments used by oncologists to support their advice (change or no change); O = arguments mentioned by oncologists that did not match their advice

*Abbreviation*: *SPA* standardized patient assessment

#### Arguments for recommending change

##### Overall

In consultations in which oncologists recommended a change in lifestyle behavior (*n* = 21), most used one to three arguments. Two oncologists did not provide any arguments to back up their advice.

##### Setting and substance

Conversations about substance use behavior were more extended in the curative setting, e.g., more arguments supporting the advice were used compared to the palliative setting (*n* = 29 vs. *n* = 14), particularly in conversations that concerned smoking (*n* = 22 arguments). 

When﻿ examining the arguments for a change in substance use behavior in the curative setting (*n* = 16), the most frequently used argument was that a change would reduce the risk of complications or side effects (*n* = 9/16, Table 2, Q2). This argument was exclusively used to back up a recommendation to quit smoking. Other arguments for a recommended change in the curative setting were that substance use behavior is unhealthy in general, irrespective of cancer (*n* = 4/16), and that such behavior negatively affects the patient’s general condition during treatment (*n* = 6/16). Four oncologists (*n* = 4/16) argued that specifically smoking may reduce the effectiveness of treatment, some explaining how it narrows the blood vessels which may hinder the effect of treatment (Table 2, Q3). Two oncologists in the curative setting suggested that the diagnosis of cancer may be perceived as an opportunity to decide to quit smoking (*n* = 2/16). For alcohol use specifically, oncologists argued that treatment may affect the patient’s taste or appetite (*n* = 3/16, Table 2, Q4). 

When examining the arguments for a change in substance use behavior in the palliative setting (*n* = 5), most oncologists argued that specifically smoking is generally unhealthy (*n* = 2/5) or that the patient’s taste of and appetite for alcohol is likely to change (*n* = 2/5); one argued that smoking may magnify the side effects of chemotherapy (*n* = 1/5).

#### Arguments for not recommending change

##### Overall

In consultations in which oncologists did not recommend a change in substance use behavior (*n* = 18), about half (*n* = 8/18) provided either one or two arguments for not recommending such a change. In many consultations in which no change was recommended, oncologists simply stated the behavior was “not a problem” or there was “no objection.”

##### Setting and substance

Of the oncologists in the curative setting who did not recommend change (*n* = 3), only one provided an argument (i.e., alcohol will not impact the effectiveness of treatment; *n* = 1/3). Of the oncologists in the palliative setting who did not recommend a change (*n* = 15), many stated that the primary aim was maintaining patients’ quality of life (see Q5), which could include smoking or using alcohol (*n* = 7/15). A few oncologists argued that smoking would not negatively impact the effectiveness of treatment (*n* = 2/15). Sometimes, oncologists who did not recommend a change (*n* = 3/15) started out by saying substance use was unhealthy in general (Table 2, Q6), and many added that patients’ taste or appetite may change as a consequence of treatment (*n* = 7/15, see Table 4). Four oncologists in the palliative setting did not recommend change, but argued that substance use may negatively impact the effectiveness of treatment or patient’s condition during treatment or that it may increase the risk of complications or side effects (*n* = 4/15).

### Support for change in substance use behavior

Nine oncologists offered some kind of support for the substance use behavior change they recommended, mostly in the curative setting (*n* = 8/9). Most of the support offers (*n* = 8/9) were related to smoking cessation. One oncologist mentioned the possibility for support with moderating alcohol use, albeit quite general (“support is available”).

When offering support, most oncologists mentioned the possibility of a referral, either to the general practitioner or practice nurse (*n* = 2/9), to a smoking cessation outpatient clinic or program (*n* = 2/9), or both (*n* = 2/9, Table 2, Q7).

Other oncologists (*n* = 2) offering support did so more generally by mentioning the fact that the patient was not alone in changing their behavior. They emphasized that the patient could count on their help or even that they were “in this together”(Table 2, Q8).

### Communication

#### Phrasing of recommendation

There was variation in how strong oncologists phrased their recommendations. Some oncologists, usually in the curative setting, had a strict tone of voice when providing recommendations about their substance use behavior, using words like “plea” and “must.” Concerning smoking, some oncologists seemed to make explicit use of their medical authority (Table 2, Q9). Some were also making a strong appeal to patients’ own responsibility, pointing out that quitting smoking would be a way to personally contribute to a good outcome (Table 2, Q10). In contrast, other oncologists, most often in the palliative setting, formulated their sentences carefully, choosing words such as “rather not” and “maybe better.” Sometimes, this carefulness resulted in ambiguous messages (Table 2, Q11).

#### Addressing the patient perspective

Many oncologists who recommended patients to quit smoking expressed their empathy for patients’ difficult circumstances in which quitting was considered particularly difficult. Sometimes, they did so in response to patients’ remarks about the importance of the behavior for them personally (Table 2, Q12). However, few actively inquired about the patient’s perspective on the substance use behavior, the recommended substance use change, or the support offered (*n* = 4). Overall, there was very limited dialogue in response to the patient’s question or in response to the provider’s information, recommendation, or offer of support.

## Discussion

This study on oncologists’ communication with esophagogastric cancer patients about smoking and alcohol use showed that oncologists are more inclined to advise for a behavior change in the curative than in the palliative setting. Compared to medical oncologists in the palliative setting, surgical and radiation oncologists in the curative setting were also more stringent in their communication about the patients’ tobacco and alcohol use. In the palliative setting, oncologists seem to prioritize the negative impact of a behavior change on patients’ quality of life over potential health and survival benefits. This tradeoff was made explicit in the phrasing of their recommendations, sometimes resulting in ambiguous messages. Yet, oncologists rarely explored the patient’s reaction to their recommendations and never involved the patient’s perspective in weighing the pros and cons of making favorable changes in their substance use. To provide oncologists with tools and reduce practice variation, clear guidelines for oncologists on when and how to provide unequivocal recommendations and support patients are needed, to ensure patients benefit most from their potential lifestyle changes and cancer treatment.

In the curative setting, oncologists provided clearer and more extensive advice on smoking, possibly due to stronger evidence linking smoking to outcomes compared to the palliative setting [25–29]. However, despite the more sparse evidence in the palliative setting, still 74% of medical oncologists in a previous survey study acknowledged the detrimental impact of smoking on treatment outcomes and 64% disagreed that quitting smoking would be a waste of time [40]. Hence, addressing smoking in the palliative setting might be hindered by oncologists’ hesitation to take away a pleasurable habit of patients or a fear to induce guilt or shame in the last phase of life.

Oncologists paid only limited attention to patients’ perspectives while communicating about substance use, both in the palliative and the curative setting. Few actively explored patients’ thoughts or reactions to the oncologist’s information, recommendation, or the offered support, aligning with a preference for an educational approach in communication over an explorative, coaching style, as indicated in previous literature [51]. Other explanations could be oncologists not feeling responsible for or competent in coaching patients in changing their substance use [56–58]. However, engaging with patients’ responses and perspectives may substantially improve the effectiveness of information and advice. It ensures patients’ understanding of the information, facilitates shared decision-making about behavior change, may motivate patients to change their behavior [59], and identifies the support a patient needs for successful behavior change.

Oncologists’ arguments to substantiate a recommendation to quit smoking in the curative setting were rather coherent across oncologists; almost all referred to an increased risk of complications or side effects, mostly related to surgery. Some oncologists added the negative impact on radiotherapy or chemotherapy effectiveness. As this argument might be an important consideration for patients, this practice variation is notable. Evidence suggests a general detrimental effect of smoking on prognosis; the evidence for an effect on treatment response and recurrence is less conclusive [60]. The American Society of Clinical Oncology (ASCO) smoking cessation guide for oncology providers recommends to inform about a reduction of the effectiveness of radiation therapy specifically [61], aligning with a meta-analysis indicating a decreased efficacy of radiation therapy, yet not chemotherapy [62]. Possibly, the inconclusive evidence and oncologists’ reluctance to instill responsibility or guilt in patients [56–58] may deter them from utilizing the reduced treatment efficacy argument.

Oncologists offer less strict advice regarding alcohol use compared to smoking. In line, ASCO professional guidelines are more directive for smoking (quitting) than alcohol use (moderating high-risk use) [61, 63, 65], although the World Cancer Research Fund recommends to “limit alcohol consumption, and in order to prevent cancer, not to drink alcohol at all” [64]. The greater social acceptance of alcohol [66], the belief in “responsible alcohol use” [65], and limited awareness of the relation between alcohol and cancer [67] might make conversations about moderating or quitting alcohol use less self-evident for both oncologists and patients. Nevertheless, consuming one drink per day is associated with an increased risk for esophagus squamous cell carcinoma [68].

Half of the oncologists offered some type of support for their recommended lifestyle change, mostly for smoking cessation and mostly in general remarks. Consistent with previous research showing oncologists’ strong preference for smoking cessation interventions to be managed by other health workers [57], most oncologists in this study referred the patient. Barriers to offering support reported in the literature include uncertainties about responsibility, limited time, insufficient education in behavior change interventions, and a lack of protocols or resources [56–58].

This study’s strengths are the utilization of observed instead of self-reported behavior and the heterogeneous sample, including oncologists from different disciplines and cases from both the curative and the palliative setting. This approach offered a comprehensive understanding of communication about substance use in esophagogastric cancer patients. The study’s limitations include uncertainty about the representativeness of this behavior for clinical practice, given that interactions were with patient actors, some conducted online, and given that actors were instructed to initiate discussions about smoking and alcohol, potentially deviating from actual practice [69]. Furthermore, it is unclear to what extent the results are generalizable to other cancer types, which may have different associations with smoking or alcohol use and have a different prognosis. For example, in the palliative setting, advanced esophagogastric cancer patients have a short life expectancy (75–81% 1-year mortality rate [70]), which may have led oncologists to prioritize patients’ quality of life over health benefits. Replicating these findings in diverse cancer populations is crucial, considering variations in prognosis and associations with substance use between different types of cancer.

To enhance the understanding of oncologists’ communication about smoking and alcohol use with patients, we propose a qualitative interview study, utilizing oncologists’ reflections on recorded consultations. Qualitative studies can further explore patients’ perspectives in the curative and palliative settings regarding how oncologists communicate with them about smoking or alcohol. In addition, it is important to examine consistency in messaging among multidisciplinary teams (i.e., surgical, radiation, and medical oncologists; physician assistants; and nurses).

Our findings stress the need for guidance in addressing changing substance use in oncology consultations. Following their own preferences [57], oncologists could initiate conversations about substance use, provide and back up their recommendations, and refer patients to specialized support. Pre-habilitation programs, emphasizing lifestyle optimization before starting treatment, show promise [71]. While these programs are increasingly incorporated in surgical care, their applicability to patients undergoing chemotherapy or radiation therapy may be promising and requires exploration. The integration of pre-habilitation programs in standard care pathways might facilitate conversations about smoking, alcohol use, and other health behaviors in cancer care.

This does not mean oncologists have a minor role. Research shows oncologists’ advice increases the likelihood of successful behavior change [35, 36, 63, 72]. Oncologists might benefit from education on the evidence linking substance use to treatment response, survival, and quality of life. In addition, such educational interventions could include scripts for effective conversations about smoking or alcohol, like the 2012 Tobacco Cessation Guide for Oncologists does [61]. Our study highlights oncologists’ limited attention to patients’ perspectives and responses. Therefore, we provide some example phrases in Box 1, which might facilitate providing tailored information, engaging in shared decision-making, and fueling a motivational conversation, potentially increasing the chances of success.

**Box 1:**
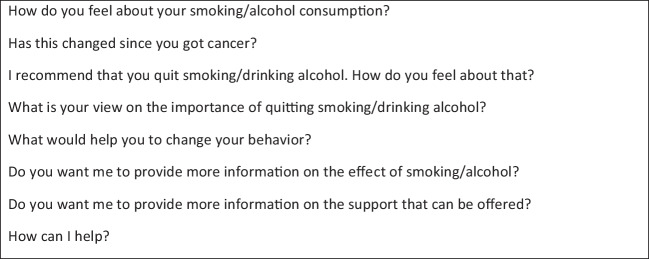
Suggested phrases for starting a conversation about smoking or alcohol use

## Supplementary Information

Below is the link to the electronic supplementary material.

Supplementary file1 (DOCX 23 kb)

## Data Availability

No datasets were generated or analysed during the current study.
