# Supplementary material for: Oncologists’ communication about tobacco and alcohol use during treatment for esophagogastric cancer: a qualitative observational study of simulated consultations
Source: Support Care Cancer. 2024 Sep 20;32(10):676. doi: 10.1007/s00520-024-08847-y (PMC11415438; doi:10.1007/s00520-024-08847-y)
Supplement: Supplementary file 1 — Supplementary file1 (DOCX 23 kb) [file 520_2024_8847_MOESM1_ESM.docx]

**Supplementary File 1. Patient cases in standardized patient assessments (SPAs)**

|  | **Palliative setting** | | **Curative setting** | |
| --- | --- | --- | --- | --- |
|  | **Case 1** | **Case 2** | **Case 1** | **Case 2** |
| **Sex** | Male | Male | Male | Male |
| **Age** | 58 | 63 | 76 | 74 |
| **Occupation** | Accountant | Retiree | Retiree | Retiree |
| **Type of cancer** | Metastasized gastric | Metastasized esophageal | Localized esophageal | Localized esophageal |
| **Indicated treatment** | Palliative systemic treatment | Palliative systemic treatment | Curative treatment | Curative treatment |
| **Substance and use** | Alcohol, 5-6 glasses a day in the weekends | Smoking, half a pack of cigarettes a day | Alcohol, 5-6 glasses a day in the weekends | Smoking, half a pack of cigarettes a day |
| **Comorbidity** | Hypertension |  | Hypertension; heart attack (10 years ago) | Hypertension; type 2 diabetes; arthrosis |

*The same three actors took on the patient roles in these four cases
